# Self-Assessment Tool to Promote Organizational Health Literacy in Primary Care Settings in Switzerland

**DOI:** 10.3390/ijerph17249497

**Published:** 2020-12-18

**Authors:** Saskia Maria De Gani, Daniela Nowak-Flück, Dunja Nicca, Dominique Vogt

**Affiliations:** 1 Health Literacy Division, Careum Foundation, 8032 Zurich, Switzerland; daniela.nowak@careum.ch (D.N.-F.); dominique.vogt@uni-bielefeld.de (D.V.); 2 Epidemiology, Biostatistics and Prevention Institute, University of Zurich, 8001 Zurich, Switzerland; dunja.nicca@uzh.ch; 3 Interdisciplinary Centre for Health Literacy, University of Bielefeld, 33501 Bielefeld, Germany

**Keywords:** health literacy, health literate organizations, primary care settings, health information, self-assessment

## Abstract

Dealing with health information and taking care of one’s own health are key aspects of health literacy and a difficulty for nearly half of the population in Europe. Limited health literacy often results in poorer health outcomes. Health literacy is a fundamental health determinant, and its improvement provides great potential for addressing public health challenges. Health care organizations play an important role in improving population’s health literacy. Health literate health care organizations facilitate access, understanding and use of health information and decrease the demands and complexities of the health care system. Few efforts have been taken so far to promote organizational health literacy, especially in German-speaking countries. This project aimed at developing a self-assessment tool, which enables primary care organizations to assess and improve their level of health literacy. The self-assessment tool was developed and evaluated with general practitioners and community care organizations in Switzerland. Here the participative development process, outcomes and the three modules of the self-assessment tool are presented: (1) manual with detailed introduction and instruction, (2) checklist for self-assessment of organizational health literacy and (3) handbook with measures for improvement. The aim of this tool is that organizations are able to identify the need for action, plan and implement improvement measures.

## 1. Introduction

Nowadays, people ought to actively take care of their own health and make informed decisions. This requires accessible, easy to understand and reliable health information, which is also tailored to people’s needs [1]. The corresponding necessary knowledge, motivation and skills—including accessing, understanding and appraising health information—have been conceptualized as health literacy. Hence, personal health literacy is “the degree to which individuals have the ability to find, understand, and use information and services to inform health-related decisions and actions for themselves and others” [2]. In this respect, it must be underlined that health literacy is always content and context-specific. This means that the ability to deal with health information is dependent on the context in which they are to be applied in order to make health-related decisions [3].

More than half of the population in German-speaking countries report low health literacy, meaning people have difficulties dealing with health information. Low income, low education, increasing age, chronic diseases, to mention a few, are related to limited health literacy [4]. Furthermore, low health literacy results in more hospitalizations and greater use of emergency care and, consequently, is associated with worse health outcomes [5]. Health literacy is, therefore, an important health determinant [6] that needs to be strengthened.

Most approaches for improving health literacy so far focus on personal health literacy by increasing people’s knowledge or specific skills [7]. However, to improve health literacy effectively, interventions need to be focusing equally on reducing the demands and complexities of the health care system. The need for organizations to better respond and act on the health literacy requirements of individuals has been recognized [6,8,9]. This approach is addressed by the so-called concept of health-literate (health care) organizations [9] or organizational health literacy, which is “the degree to which organizations equitably enable individuals to find, understand, and use information and services to inform health-related decisions and actions for themselves and others” [2].

Health and social care organizations are able to empower the population they serve by ensuring health-literate friendly and responsive structures and processes. Health literate health care organizations facilitate access, navigation, understanding and use of health information and services for the population to take care of their own health [8]. These organizations can be characterized by the following attributes [8]: They approve health literacy as an organizational value and integrate it in their structure, process and culture; they aim to strengthen health literacy of their users, their staff, as well as on different interfaces; they facilitate access to and navigation within their organization; they use clear and easy to understand oral and written communication, and they meet the needs of their users. Health care organizations that internalize these attributes enable their users to access and effectively use health services and materials, increase their competencies, and substantially contribute to better health outcomes. These attributes have been used to conceptualize organizational health literacy in organizational assessment tools and guides to support the implementation thereof in health organizations [10,11].

However, until today only a few tools have been developed to assess organizational health literacy and to support organizations to increase health literacy successfully. One of them is the “self-assessment tool to investigate the organizational health literacy of hospitals” [12,13] following the “Vienna Concept of Health Literate Hospitals and Health care Organizations” (V-HLO), which is predominantly tailored towards hospitals. A tool that focuses on community service organizations is “HeLLO Tas!” [14], a Tasmanian toolkit for developing organizational health literacy. Another tool from the southern hemisphere is the “Organizational Health Literacy Responsiveness (Org-HLR) self-assessment tool”, which addresses a broad range of organizations across the health and social service sector [15]. A tool from Germany assesses which attributes of health literate health care organizations are implemented in certain health care organizations [16]. Furthermore, there are two tools from the United States that aim at improving health literacy in hospitals [17] and health care settings [18]. Such self-assessment tools have shown to be useful in supporting monitoring, continuous quality improvement activities, self-reflection, organizational learning and benchmarking in order to enhance organizational performance and effectiveness [19]. Despite the huge potential of such tools to assess and enhance organizational health literacy, they need to be specific in order to be successful in practice. Most approaches have merely focused on inpatient care or health care organizations in general; specifically, outpatient and/or primary care settings have marginally been integrated. However, especially primary care settings offer a huge opportunity to enhance health literacy of the population, as they are often the first and a regular contact within the health system.

Only recently, the concept of health literate organizations has gained attention in Switzerland. Hence, there is a lack of comprehensive tools and interventions to improve organizational health literacy in health care settings in general, but especially in primary care settings [10,20]. As mentioned above, particularly primary care settings with their regular and close contact with their users have great potential in increasing their health literacy levels. Therefore, there is an urgent need to design, implement and evaluate tools in respect to effectiveness that enables organizations to become a health literate health care organization. By strengthening the health literacy of health service providers, especially in primary care, the health literacy of the population can eventually be enhanced.

The aim of this implementation-oriented research project was to close the research gap by developing and evaluating a self-assessment tool for general practitioners and community care services to improve the health literacy of primary care organizations in Switzerland. Based on already existing tools [8,13,14,15] and in a participative process with general practitioners and community care service organizations, our objective was to develop a tool that assesses the current situation of a primary care organization, supports the organization to create an action plan and provides information for implementation of the defined actions. Therefore, we developed a tool called “Organizational Health Literacy Self-Assessment Tool for Primary Care” (OHL Self-AsseT) for primary care settings in Switzerland, consisting of the following three modules: (1) a manual containing a clear and easy to understand introduction into the health literacy concept and detailed instructions on how to use the tool; (2) a checklist for the self-assessment of general practitioners and community care services in Switzerland; (3) a handbook as a reference for measures of improvement according to the content of the checklist. This article focuses on the checklist that builds the core of this self-assessment tool. The participative development process of the self-assessment tool, together with the outcomes, is presented in short as well. The three modules of the self-assessment tool are described, and the entire translated checklist is shown in the results part.

## 2. Materials and Methods

This project was organized into two parts. The first part served to generate the structure, domains and content of the self-assessment tool and to create the first draft. In order to develop a self-assessment tool for general practitioners and community care services, we performed a literature review of existing tools and interventions in the field of organizational health literacy. Combining the results of the literature review and in constant exchange with our practice partners and experts from Austria (Gesundheit Österreich GmbH), we developed a draft of the self-assessment tool. In the second part, we conducted group and expert interviews to evaluate the draft and revised the self-assessment tool accordingly. In the current method section, we describe not only the development process itself (literature review, draft, evaluation with interviews) but also the results of the evaluation interviews and the revision of the draft. The self-assessment tool consisting of the three modules (manual, checklist, and handbook) is then described in the results section.

### 2.1. Narrative Literature Review and Adaption of Existing Tools

In a first step, we conducted a narrative literature search in online databases (PubMed, CINAHL, PsychInfo), which was complemented by extensive hand searches. The aim of the literature search was to obtain a comprehensive insight into the topic of organizational health literacy as well as available instruments and materials. Since this was not a systematic search but rather a collection of material, no detailed bibliographic information can be provided. For this literature search, we used the following keywords: health literacy, organization, environmental health literacy, health literacy responsiveness, health literate health care organization, organizational health literacy, improving. The keywords were combined with AND or OR and formed into a search term. Publications were included for further screening when they related to health literacy at an organizational level and were published in English or German language. No limits of publication date or study design restrictions were made. Afterward, the identified publications were screened and then analyzed with regard to their relevance to the study objective. The analysis of the literature focused on identifying conceptual papers on organizational health literacy as well as assessment tools and health literacy guides. The results were listed in a table for internal project work and were processed by content analysis.

To date, very few tools in the field of health literacy of health care organizations exist [10]. Accordingly, we identified two tools as relevant to inform the development of our checklist: First, we thoroughly examined the comprehensive, piloted and validated “self-assessment tool to investigate the organizational health literacy of hospitals” [13], following the V-HLO. The V-HLO linked organizational health literacy to quality management and drew on models of quality improvement and organizational development. The according tool comprises 9 standards, 22 sub-standards and 160 items in German language. The checklist is recommended to be completed by a multidisciplinary team of workforce members that are in charge of the quality of the organization [13]. The tool has lately been adapted to an international version in English language and one that covers different health care settings [12]. Second, we used the Tasmanian toolkit for developing organizational health literacy “HeLLO Tas!” [14] to inform the development of our checklist. This toolkit consists of three parts: (1) the process (to become a health literate organization), (2) the checklist (to assess the actual status of the organization) and (3) the tools (to support the development process). This intuitive structure inspired us to use a similar composition of three modules (manual, checklist and handbook). Additionally, “HeLLO Tas!” describes a framework of six dimensions of a health literate organization, which are: access and navigation, communication, consumer involvement, meeting the needs of diverse communities, workforce and leadership and management. Moreover, the Tasmanian self-assessment checklist has a clear and remarkable design, and its items are written concisely and in plain language. Accordingly, we also tried to develop a clear design and write everything in an easily understandable language.

### 2.2. Development of the Draft

According to our literature search and in discussion with our practice partners, we defined the structure of our self-assessment tool. We decided to organize the tool into three modules: (1) a manual, (2) a checklist and (3) a handbook.

#### 2.2.1. Manual

The manual mainly contains instructions on how to use the checklist and guides the users step-by-step through the self-assessment process.

#### 2.2.2. Checklist

Based on the ten attributes of a health literate health care organization [8], the nine standards of the V-HLO tool [12], the framework containing six dimensions of a health literate organization used by the Tasmanian toolkit [14] as well as in close discussion with our practice partners (representatives of general practitioners and community care organizations), we elaborated the dimensions, sub-dimensions and criteria defining a health literate primary care organization in Switzerland. Table 1 shows an example of the development of one dimension using the above-mentioned resources.

Accordingly, we defined the following six dimensions: “provide easy access to primary care service and facilitate navigation within”, “communicating in plain and easy to understand language”, “promoting health literacy of our users”, “promoting health literacy of staff members”, “incorporating health literacy into the management and organizational structure” and “promoting health literacy at care interfaces, networks and further activities of the organization”. These six dimensions include and aggregate the most important attributes, standards or dimensions of health literate organizations according to our literature research as well our discussions with practice partners and our colleagues from Austria (Gesundheit Österreich GmbH), as they were conducting a similar project. Consequently, the present six dimensions represent crucial characteristics of health literate primary care organizations and are specifically tailored towards primary care organizations, which was one of our major concerns. At the same time, we aimed at developing dimensions, which are concise and clear. This means that the dimensions should be clear for all workforce members of primary care settings, important for their everyday work as well as their organization’s development process. Moreover, they should be able to fill in the checklist within a reasonable time.

These dimensions formed the main content of the checklist and were further divided into sub-dimensions and criteria. To give an example: For the first dimension, “provide easy access to primary care service and facilitate navigation within”, we defined the two sub-dimensions “contact” and “navigation within the primary care service”. Each sub-dimension then consists of multiple criteria, which are the statements the workforce members are rating and which are used to assess the primary care service organization (see results, Table 2). We adopted the rating scale for our checklist from the Vienna model [12] defining the following four categories for the degree of fulfillment: not fulfilled (0–25%), rather not fulfilled (26–50%), rather fulfilled (51–75%) and completely fulfilled (76–100%). According to our practice partners, these categories seem intuitive to be answered. Besides these four categories, we also added the option to indicate that a specific criterion is “not applicable”. Moreover, we left some space for remarks. To support the comprehensibility and for clarification purposes of each criterion, we worked out specific examples. Considering the different challenges, situations and circumstances of general practitioners and community care services, an issue that clearly emerged from our discussions with the practice partners, we developed two versions of the checklist to address these differences, especially concerning the specific examples for each criterion. Thus, the checklist for general practitioners specifically addresses general practitioner workforce members. Moreover, the examples given in order to ease comprehension of each criterion were tailored towards general practice. As a specific explanatory example, we used the term “treatment plan” for general practitioners and “care plan” for community care service. However, in order to be able to publish a tool for primary care services in general, the present checklist (see results, Table 2) applies more general terms.

#### 2.2.3. Handbook

According to the dimensions, sub-dimensions and criteria of our checklist, we developed the handbook. The handbook is based on existing German [21], Austrian [22] and Swiss [23] tool-boxes for health literate organizations and refers to a few other mainly national offers and opportunities when useful. The purpose of the handbook is to support the organization’s improvement process to become more health literate by describing a set of ideas, indications, strategies, measures as well as instruments. The idea after applying the checklist is to identify strengths and weaknesses and define the need for action. At this point in the organization’s development process, the handbook assists organizations in planning and implementing improvement actions specific to the criteria that, according to the self-assessment, were met to a lesser degree.

### 2.3. Evaluation Interviews

After the development of the draft, containing the three modules manual, checklist and handbook, we evaluated the user-friendliness, content and applicability of it. Therefore, we recruited members of our practice partner organizations (representatives of general practitioners and community care organizations) and primary care experts from other organizations. They received our tool with an accompanying information letter to study it thoroughly before being interviewed. For these evaluation interviews, we recruited 3 care specialists, 6 qualified nurses and nursing assistants and 4 housekeepers from the community care organization. Regarding our partners from the general practitioners, we were able to recruit 1 practice manager and 1 medical doctor. Thereafter, we organized four group interviews with these experts (two interviews with teams of the community care organization and two interviews with the general practitioners). In addition, we conducted four expert interviews with primary care experts from other organizations (2 medical doctors and 2 leading care and support specialists).

The interviews were conducted in a semi-structured way and followed a specifically developed guideline to allow for systematic feedback. We asked for feedback on each module separately. The questions targeted aspects such as comprehensibility, the process of the assessment, relevance of the dimensions, completeness of relevant criteria, accurate examples, practical relevance, usability, user-friendliness and general impression. Here are some example questions: “How relevant is the content of the manual/checklist/handbook?”, “If you were given the manual and the checklist to perform a self-assessment, would you need any additional information?”, “Are the dimensions presented in the checklist understandable?” and “How do you rate the length of the checklist? Is there content missing or that could be left out?”. All interviews were recorded and logged.

### 2.4. Results of Evaluation Interviews

The results of the interviews were collected, categorized and processed in one spreadsheet. Subsequently, we examined the condensed feedback and implemented valuable indications and suggestions into the here presented self-assessment tool (manual, checklist and handbook). Overall, the tool received very positive feedback in the interviews. Briefly, the specific and clear directions, as well as the explanatory graphics in all of the modules, were appreciated. The clear instruction of the manual and the recommendation of building an interprofessional self-assessment-team to consider different opinions within the organization were valued. On the other side, difficulties were mentioned in understanding the theoretical part of the manual, wherefore we rewrote that part in a more comprehensible language. Furthermore, the comprehensiveness of the criteria and the consideration of the two settings in the checklist were appreciated. The interview partners highlighted the clear and accurate examples in the checklist, which were given for each criterion. Despite being slightly overwhelmed by the thoroughness of the criteria, the participants did not recognize any shortening or downgrading potential and considered the comprehensive list of criteria inevitable. In order to successfully implement and distribute the tool, the interview partners emphasized the commitment of the organization’s management and the provision of incentives, therefore. As every organizational development process is dependent on the management’s commitment, we considered this aspect more specific in the manual as well as the handbook.

### 2.5. Translation of the Checklist

To provide the checklist also for primary care organizations in non-German-speaking countries and other interested parties, we translated the final version of our checklist into English. Thereby, we matched our translation (language, but not content) with the already existing tools in English language [13,14].

## 3. Results

The self-assessment tool called “Organizational Health Literacy Self-Assessment-Tool for Primary Care” (OHL Self-AsseT) consists of the following three modules: (1) manual, (2) checklist, (3) handbook. The self-assessment tool (including the checklist) has originally been developed in German language.

### 3.1. Manual

The manual gives a brief introduction to the topics of health literacy, health literate organizations as well as organizational development in general. Aside from the theoretical introduction, the manual mainly focuses on detailed and clear instructions on how to perform the self-assessment. Briefly, the manual includes how to evaluate the current level of health literacy of a particular primary care organization or network and how to proceed from thereon.

First, it is recommended to conduct the self-assessment in consultation with the organization’s management and to decide whether the assessment is performed for the team only or for the possible corresponding organizations as well. An interprofessional team of at least two workforce members is recommended to undertake the self-assessment. This procedure allows the integration of different perspectives within the organization. This so-called self-assessment-team can be built separately or be represented by an already existing quality management team. Moreover, we recommend designating a responsible person for the coordination of the self-assessment process.

**Figure 1:**
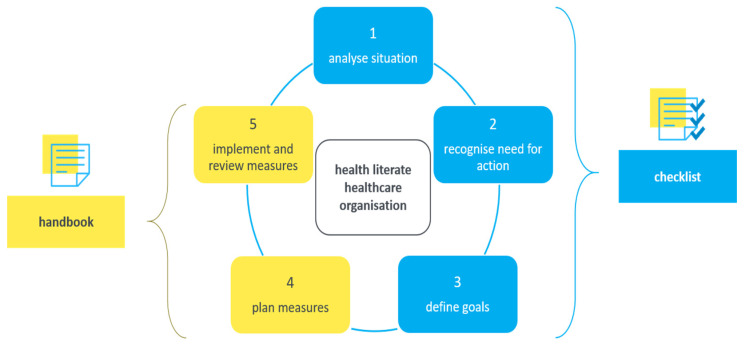
Stepwise organizational improvement process.

As quality improvement processes always proceed stepwise, the manual describes five steps to evolve towards a more health literate health care organization (Figure 1). The second module (checklist), together with the enclosed material (see below), thereby covers steps 1 to 3, which can be conducted in one or two meetings of around two hours total time required. The idea is that every member of the self-assessment-team fills in the checklist according to his or her own personal opinion. Thereafter, the self-assessment-team discusses their individual ratings and together reaches a consensus for their entire organization (step 1). Based on the consensus, the team identifies strengths, weaknesses and needs for action (step 2) and defines one to three development goals (step 3). Thereupon, the self-assessment-team reviews the development goals, discusses and defines one or two improvement measures per goal (step 4). In this process, the handbook assists them. This step takes one or two meetings of approximately one hour in total duration. Additionally, the defined measures need to be coordinated with the management. In the last step, the organization implements and regularly reviews the endorsed measures (step 5). The time required for implementing and reviewing defined measures cannot be indicated, as this depends on the organizational circumstances. In order to get a complete overview of the current level of organizational health literacy, it is recommended to check all the dimensions of the tool. However, as the tool is comprehensive but also modularized, it can be used either for a total organizational assessment or for an assessment of a specific aspect of organizational health literacy.

### 3.2. Checklist

To consider the heterogeneity of primary care settings, originally, two separate questionnaires were developed, one for general practitioners and one for community care services. However, as mentioned above, here, we present the generic version (Table 2). Furthermore, the tool considers different organizational structures, such as whether a general practitioner or a care team belongs to a head organization (see checklist dimensions 5 and 6).

### 3.3. Handbook

The handbook describes a set of ideas, indications, strategies, measures and instruments to support primary care settings in Switzerland in improving aspects of organizational health literacy. The handbook is based on the structure of the checklist (Table 2). However, there is no separation between the two settings, general practitioners and community care service. Each chapter starts with a short introduction on the relevance and the practical application of the corresponding dimension and is followed by descriptions and links to specific measures, instruments or training. The handbook intends to be a reference and does not guarantee to be complete, as it only represents a range of instructions and methods that were evaluated for quality and reliability. Moreover, it only refers to non-commercial providers and offers and recognized public institutions.

## 4. Discussion

The aim of the article was to present a self-assessment tool, which was developed to assess and improve organizational health literacy in primary care organizations in Switzerland. Considering the lack of comprehensive tools and interventions to evaluate and strengthen organizational health literacy in primary care settings [10,20], this “Organizational Health Literacy Self-Assessment-Tool for Primary Care” (OHL Self-AsseT) provides a practice-oriented intervention for primary care organizations to independently assess and improve their organizations’ health literacy. The self-assessment tool consists of three modules: (1) manual, (2) checklist (Table 2), (3) handbook. These three modules not only enable primary care organizations to independently perform a self-assessment but also include guidance in improving health literacy within their organization as well as of their users. To ensure practice relevance, the present self-assessment tool has been developed in collaboration with various experts in primary care settings.

In accordance with the validated, comprehensive V-HLO tool [13], the dimensions, sub-dimensions and criteria of the OHL Self-AsseT consider a broad understanding of health literacy [6,24] and focus on developing organizational structures and processes to improve health literacy sustainably. With the objective of providing a concise and application-oriented tool and with respect to our literature review, we condensed the ten attributes of health literate health care organizations [8] or the nine standards of the V-HLO [13], respectively, into six dimensions specific for primary care organizations. Thus, the checklist (Table 2) resulted in a concise tool that supports primary care organizations in promoting health literacy.

In contrast to the V-HLO tool, but in accordance with the Tasmanian tool [14], the present tool not only provides an assessment of health literacy but supports organizations with an according handbook to systematically improve organizational health literacy. This handbook is consistent with the more up-to-date health literacy toolkits [10] that provide only evidence-based recommendations and best practices to implement health literacy promoting measures. Moreover, the handbook reflects the dimensions of the self-assessment and facilitates the application of the tool. However, despite considering the practical level during the development of the entire self-assessment tool, its applicability in organizational practices has not been tested so far.

### 4.1. Future Directions

To verify practical feasibility and effectiveness, we will apply the self-assessment tool in four general practitioners and eight community care teams in the canton of Zurich over a period of six months. For detailed evaluation in terms of user-friendliness, usability, usefulness and potential development processes towards a more health literate organization, focus groups with interprofessional workforce members and expert interviews with leaders of those organizations will be conducted. Moreover, a standardized questionnaire assesses the development of knowledge in and attitude towards health literacy of the entire workforce of these pilot companies. Based on the evaluation results, we will adapt the self-assessment tool one more time and provide all materials free of charge. The results of this practical evaluation will be published later.

Once the self-assessment tool is approved in everyday practice, it still needs to be ensured that primary care organizations make efforts to increase their organizations’ health literacy and independently apply the tool. Thus, we are fully aware of the need to provide incentives so that general practitioners and community care organizations voluntarily apply and implement the self-assessment tool. Therefore, in a further step, we will be working on an implementation strategy of the present self-assessment tool in order to accomplish sustainable changes and development processes in primary care organizations. A possible relevant framework to successfully implement the OHL Self-AsseT in primary care settings that could be considered during this process is the famous “behavior change wheel” [25]. Important steps in this direction and the involvement of relevant stakeholders are necessary for a successful future of this field.

### 4.2. Limitations

We acknowledge that the development of the present OHL Self-AsseT is not without limitations. Ideally, a larger cohort of users could have been involved in the development and evaluation of the tool—specifically, in terms of our group and expert interviews. However, we used our resources to the maximum, and with our future directions, we are confident to be able to further optimize our tool.

## 5. Conclusions

Conclusively, the present tool adds to the development of health literate health care organizations by a comprehensive package of tools (manual, checklist (Table 2) and handbook), including assessment and interventions for primary care needs specifically. The participative development with practice partners and experts has shown great acceptance and, therefore, may support successful implementation in primary care settings. In order to scale the implementation of the present “Organizational Health Literacy Self-Assessment-Tool for Primary Care” (OHL Self-AsseT) up, an evaluation of its application in several pilot companies is planned. The evaluated and refined self-assessment tool could serve as a powerful instrument to improve organizational health literacy internationally, not only in primary care settings in Zurich or Switzerland.

Nonetheless, a future systematic implementation thereof is necessary to improve health literacy system-wide. Therefore, organizational health literacy needs to be considered on the political agenda. Furthermore, it is also important to integrate health literacy in the education and training of health care professionals.

## Figures and Tables

**Table 1:** Example of a dimension’s development.

Dimensions of the OHL Self-AsseT	Attributes of a Health Literate Health Care Organization [8]	Standards of the V-HLO [12]	Dimension of “HeLLO Tas!” [14]
2. Communicating in plain and easy to understand language.	6. Uses health literacy strategies in interpersonal communications and confirms understanding at all points of contact. 8. Designs and distributes print, audiovisual, and social media content that is easy to understand and act on. 9. Addresses health literacy in high-risk situations, including care transitions and communications about medicines. 10. Communicates clearly what health plans cover and what individuals must pay for services.	2. Develop documents, materials and services with stakeholders in a participatory manner	Communication
		5. Apply health literacy best-practices in all forms of communication with patients	

**Table 2:** “Organizational Health Literacy Self-Assessment Tool for Primary Care” (OHL Self-AsseT).

**1. Provide easy access to primary care service and facilitate navigation within**	
Contacting our primary care service is possible in a simple way for users. Access to our buildings and premises is clearly visible. Navigation within the primary care service is easy.	
1.1 Contact	
1.1.1	We offer **several ways** for our users to contact us (phone, email, website).
1.1.2	Our phone numbers, addresses and our website are **clear and easy to find** in directories (e.g., internet, information brochures).
1.1.3	Our website is **user-friendly and easy to understand** even for people with poor digital competencies as well as for people with physical and cognitive disabilities (e.g., use of plain language, adjustable font size, available color choice, simple navigation, read-aloud function).
1.1.4	We **react appropriately to questions** from our users on the phone, through email or at the main entrance.
1.1.5	We offer **easily accessible and understandable information about our location** and the journey to our primary care service.
1.2 Navigation within the primary care service	
1.2.1	**The building and the entrance** of our primary care services are clearly marked and visible (e.g., with signs, indications).
1.2.2	**The individual areas** within our primary care service are clearly marked and visible (i.e., reception, waiting area, consultation room, meeting room, washrooms).
**2. Communicating in plain and easy to understand language**	
Oral and written communication with our users is based on health literacy standards.	
2.1 Oral communication	
2.1.1	We create circumstances that allow **calm communication** (e.g., relocate to an appropriate room, closing doors).
2.1.2	We dedicate **sufficient time** for conversations with our users.
2.1.3	We use **plain language** in a conversation with our users (e.g., when explaining the intake of medication or clarifying technical terms).
2.1.4	In conversations with our users, we ensure the **information given is understood** (e.g., through conversation techniques such as teach-back).
2.1.5	We explicitly **encourage** our users **to ask questions** or to express any concerns.
2.1.6	We provide **written notes** on important information and **key messages** from the conversation with our users if required.
2.1.7	We respond to **different needs** and language requirements of our users (e.g., through mother tongue assistance, visual material and pictograms).
2.1.8	We have **guidelines** for
	a. verbal communication, which follows health literacy best practices (according to questions 2.1.1–2.1.7),
	b. communicating in risk situations (e.g., communicating bad news, preparation for surgical interventions, new therapies).
2.2 Written communication	
2.2.1	We use **plain language** in our written materials and information (e.g., in information sheets, forms).
2.2.2	We design **clear and easy-to-understand** written material and information (e.g., by using appropriate font size, line spacing, color contrast, images).
2.2.3	We provide and recommend **material and resources** (e.g., brochures, digital applications) that are:
	a. up to date,
	b. technically correct,
	c. available in the mother tongue of the larger user groups.
2.2.4	We provide **assistance** for our users in **completing forms** (e.g., in case of referrals, registration, patient decree).
2.2.5	We have **guidelines** for written communication, which take into account the above-mentioned aspects of communication following health literacy best practices (question 2.2.1–2.2.4).
**3. Promoting health literacy of our users**	
We enhance health literacy of our users and support them to be experts of their well-being as well as to cope with chronic conditions.	
3.1 Empowering our users to use health information	
3.1.1	We empower our users
	a. **to access health information** (e.g., by referencing good and reliable sources of information, brochures, links, contact person),
	b. **to appraise health information** (e.g., through explanation, replying to inquiries),
	c. **to evaluate health information** (e.g., through informing and explaining different options and their advantages and disadvantages),
	d. **to apply health information** to make informed decisions in regards to their own health (e.g., decisions regarding diagnostic methods and therapies, changes in lifestyle).
3.2 Promoting an active role and self-management of our users	
3.2.1	We **provide information** to our users about:
	a. **the treatment schedule/care plan,**
	b. possible ways they **can contribute to coping with their condition,**
	c. their contribution to their **mental and physical health.**
3.2.2	We offer **courses** to our users about the following topics, or we refer them to other adequate providers:
	a. **coping with chronic disease** (self-management),
	b. **lifestyle changes** (e.g., nutrition and exercise, health coaching, stop smoking),
	c. **use of health information and conversational skills** (e.g., how to find trustworthy health information, contributing to a good and informative conversation with a health professional).
**4. Promoting health literacy of staff members**	
Enhancing health literacy of our users is part of our staff members’ professional competence. Health literacy is part of the personnel development. Note: The following questions are particularly for staff members in direct contact with users.	
4.1 Know-how and professional competence	
4.1.1	We as staff members know
	a. the meaning of **health literacy** (note: see instruction for a definition),
	b. how to **enhance the health literacy** of our users (e.g., provide trustworthy information, simple and easy-to-understand communication, promoting self-management competences),
	c. where to find **good and reliable information** for our users (e.g., about symptoms, diagnostic methods, therapies, guidelines of the health system).
4.2 Personnel development	
4.2.1	We receive **training and/or materials** to build and extend our knowledge of health literacy.
4.2.2	We receive training in **health literate communication:**
	a. the use of plain language (no jargon and technical terms, simple sentences),
	b. active listening and how to stimulate questions being asked,
	c. the use of reconfirmation techniques to ensure our users have understood the context of the conversation (e.g., Chunk-and-Check, Teach-Back),
	d. supporting conversations with written and audiovisual tools,
	e. dealing with users speaking a foreign language,
	f. motivational interviewing,
	g. communicating in risk situations.
4.2.3	We receive training and/or materials about how to support our users:
	a. **to cope with common chronic disease** (self-management),
	b. through **lifestyle changes** (e.g., nutrition and exercise, health coaching, stop smoking).
4.3 Staff members’ health	
4.3.1	Staff members are supported in **their personal health literacy** (e.g., through training):
	a. dealing with professional health risks,
	b. lifestyle changes.
**5. Incorporating health literacy into the management and organizational structure**	
Health literacy is part of the management principles of our organization and is embedded in the structure, processes and culture of our primary care service team. Health literacy is a development goal of our primary care organization. We collect feedback from our users to issue and refine documents and services. Note: Depending on how your primary care team is organizationally embedded, you may only be able to answer questions for your primary care team and/or also for your primary care organization.	
5.1 Health literacy as an organizational responsibility	
5.1.1	In our **strategic documents**, health literacy is defined as an organizational responsibility (e.g., in the mission statement, in policies, in business goals). Answer for
	i. primary care team
	ii. primary care organization
5.1.2	We provide **financial support** and recruit a **responsible person** to enhance organizational health literacy. Answer for
	i. primary care team
	ii. primary care organization
5.2 Health literacy as a development goal	
5.2.1	We **define and implement goals** and measures to further develop as a health literate organization. Answer for
	i. primary care team
	ii. primary care organization
5.2.2	We regularly **review** whether our **goals and measures** to further develop as a health literate organization are being reached and refine them accordingly. Answer for
	i. primary care team
	ii. primary care organization
5.3 Organizational culture	
5.3.1	**Health literacy is an important topic** for our management, and this is regularly communicated. Answer for
	i. primary care team
	ii. primary care organization
5.3.2	We are convinced that it is important to **enhance the health literacy** of our users. Therefore, we want **to further develop as a health literate organization**. Answer for
	i. primary care team
	ii. primary care organization
5.4 User involvement - feedback	
5.4.1	We gather **feedback** from our users on the following aspects, and it will be considered in the development and improvement of our services and documents: **medical care, services and processes** (e.g., making contact, access to the doctor’s office/our spaces, referrals, support services, care services, medical measures, provided information) Answer for
	i. primary care team
	ii. primary care organization
5.4.2	We gather **feedback** from our users on the following aspects, and it will be considered in the development and improvement of our services and documents: **documents, materials and resources** (e.g., brochures, forms, declaration of consent, digital applications) Answer for
	i. primary care team
	ii. primary care organization
**6. Promoting health literacy at care interfaces, networks and further activities of the organization**	
We support our users at care interfaces. Networking with external services is used to enhance health literacy of our users. The organization is active in promoting health literacy beyond its performance mandate. Note: Depending on how your team is organizationally embedded, you may only be able to answer questions for your team or also for your primary care organization.	
6.1 Care interfaces	
6.1.1	In case of a referral, we **ask** our users whether further support is needed.
6.1.2	We **support the transfer** of our users to other service providers (e.g., arranging appointments, collecting documents and filling in forms through information exchange between service providers).
6.1.3	**In between visits**, we **contact** our users in order to ensure they have understood their diagnosis, their treatment schedule/care plan and health care services and are able to implement the first steps.
6.1.4	We point out **possible further important services** (e.g., pharmacists, community care services, physicians, podiatry, etc.) to our users.
6.2 Networking and further activities	
6.2.1	**Along with other organizations and partners**, we offer and develop resources and materials to enhance health literacy of our users (courses, consulting services and information materials on how to handle health information and self-management). Answer for
	i. primary care team
	ii. primary care organization
6.2.2	We are committed to **promoting health literacy on a higher level** (e.g., supporting research and practical projects, activities to promote changes on a political level). Answer for
	i. primary care team
	ii. primary care organizations
6.2.3	**We distribute our activities and experiences** in health literacy in internal and external committees, publications, presentations, etc. Answer for
	i. primary care team
	ii. primary care organizations
